# Cardioprotective Natural Compound Pinocembrin Attenuates Acute Ischemic Myocardial Injury via Enhancing Glycolysis

**DOI:** 10.1155/2020/4850328

**Published:** 2020-10-15

**Authors:** Yanjun Zheng, Guoqing Wan, Bo Yang, Xuefeng Gu, Jingrong Lin

**Affiliations:** ^1^Shanghai University of Medicine & Health Sciences Affiliated Zhoupu Hospital, Pudong New Area, Shanghai 201318, China; ^2^Shanghai Key Laboratory of Molecular Imaging, Shanghai University of Medicine and Health Sciences, Shanghai 201318, China; ^3^Department of Hypertension, Ruijin Hospital, Shanghai Institute of Hypertension, Shanghai Jiao Tong University School of Medicine, Shanghai, China

## Abstract

**Purpose:**

Emerging evidence has shown that pinocembrin protects the myocardium from ischemic injury in animals. However, it is unknown whether it has cardioprotection when given at the onset of reperfusion. Also, mechanisms mediating the cardioprotective actions of pinocembrin were largely unknown. Thus, this study is aimed at investigating the effects of pinocembrin postconditioning on ischemia-reperfusion (I/R) injury and the underlying mechanisms.

**Methods:**

The *in vivo* mouse model of myocardial I/R injury, *ex vivo* isolated rat heart with global I/R, and *in vitro* hypoxia/reoxygenation (H/R) injury model for primary cardiomyocytes were used.

**Results:**

We found that pinocembrin postconditioning significantly reduced the infarct size and improved cardiac contractile function after acute myocardial I/R. Mechanically, in primary cardiomyocytes, we found that pinocembrin may confer protection in part via direct stimulation of cardiac glycolysis via promoting the expression of the glycolytic enzyme, PFKFB3. Besides, PFKFB3 inhibition abolished pinocembrin-induced glycolysis and protection in cardiomyocytes. More importantly, PFKFB3 knockdown via cardiotropic adeno-associated virus (AAV) abrogated cardioprotective effects of pinocembrin. Moreover, we demonstrated that HIF1*α* is a key transcription factor driving pinocembrin-induced PFKFB3 expression in cardiomyocytes.

**Conclusions:**

In conclusion, these results established that the acute cardioprotective benefits of pinocembrin are mediated in part via enhancing PFKFB3-mediated glycolysis via HIF1*α*, which may provide a new therapeutic target to impede the progression of myocardial I/R injury.

## 1. Introduction

Cardiovascular disease remains the main cause of death worldwide [1]. Currently, there are few effective drugs to protect the heart after ischemia/reperfusion (I/R) injury. Therefore, it has become a hot research field to find the molecular mechanism of coronary artery disease progression and development that can protect the myocardium from I/R injury. With this in mind, more and more attention is focused on pharmacological interventions because it can induce cardioprotection and easy to implement [2]. However, for patients with ischemic heart disease, there are still many restrictive drugs available clinically.

Pinocembrin (5,7-dihydroxyflavone) is an abundant flavonoid isolated from propolis and some plants. It has various biological functions such as anti-inflammatory, antioxidation, and antibacterial [3]. Previous studies have confirmed that pinocembrin confers neuroprotective effects during cerebral ischemic I/R [4–6]. Recent research has also tried to determine whether pinocembrin is beneficial for cardiac injury. For example, several studies suggest that pinocembrin can improve the cardiac function of myocardial I/R rats, reduce ventricular arrhythmia, and reduce the area of myocardial infarction [7, 8]. However, it is still unclear whether pinocembrin given at the onset of reperfusion has cardioprotective effects, which is a more clinically effective method. In addition, the underlying mechanism by which pinocembrin can provide cardioprotection is largely unknown.

Recent findings report that changes in energy metabolism relate to many human diseases, and targeted energy metabolism intervention may have beneficial effects on these diseases [9]. Due to the mechanical function of the heart, it is an organ with high energy requirements. In general, most of the energy (about 70%) of a healthy heart comes from the *β*-oxidation of fatty acids, and the rest of the energy comes from glucose oxidation [10]. Nevertheless, in a pathological environment, substrate utilization may change [11]. In patients with diabetes, circulating blood glucose levels at admission are related to the clinical outcome after acute myocardial infarction (AMI), suggesting that it may be related to myocardial metabolism [12]. A metabolic shift from *β*-oxidation to glycolysis metabolism will reduce the cell's need for oxygen by 11-13%, and NAD+ precursors have been shown to activate cellular glycolysis to protect the heart from ischemic injury [13]. It is worth noting that redirect energy metabolism to glycolysis can reduce oxidative damage and inhibit apoptosis [14–16]. However, few agents that target energy metabolism are clinically safe and useful for patients. At the same time, it is unclear whether and how pinocembrin regulates acute myocardial I/R glycolysis.

In this study, we (i) examine the role of pinocembrin in rat and mouse cardiac I/R injury *ex vivo* and *in vivo*, respectively, (ii) clarify the effects of pinocembrin on the glycolytic metabolism during I/R, and (iii) investigate the underlying molecular basis that contributes to the pinocembrin-induced cardioprotection. Our results uncover a novel mechanism for pinocembrin-induced cardioprotection and suggest a potential application value of pinocembrin in the protection of hearts against I/R injury.

## 2. Materials and Methods

### 2.1. Chemicals and Reagents

3-(3-Pyridinyl)-1-(4-pyridinyl)-2-propen-1-one (3PO) was purchased from EMD Millipore (MA, USA). Pinocembrin and other reagents were supplied by Sigma-Aldrich (St. Louis, MO, USA).

### 2.2. Animals

Male Sprague-Dawley rats (aged 6-8 weeks) and C57BL/6 mice (aged 8-10 weeks) were from Shanghai Slac Laboratory Animal Co. Ltd. All animals were housed in a temperature-controlled (24°C) and humidity-controlled (40-70%) barrier system with a 12-hour light/dark cycle. All animal experiments were conducted to the guidelines for the Care and Use of Laboratory Animals published by the United States National Institutes of Health (revised in 2011), and all procedures were approved by the Shanghai University of Health and Medicine. Rats and mice were selected for treatment and observed in a randomized, double-blind trial.

### 2.3. *Ex Vivo* Global I/R Injury Model

As mentioned before, the heart was rapidly excised at 37°C and perfused at a constant pressure of 80 mmHg with Krebs-Henseleit buffer (KHB) using the Langendorff technique (Xie et al., 2005). The PowerLab system (AD instrument, Australia) was used to monitor the left ventricular imaging pressure (LVDP), left ventricular imaging pressure (LVEDP), maximum rate of pressure changes over time (+dP/dtmax), and pressure decay over time (-dP/dtmax). Pinocembrin's final concentration of 10, 30, and 100 *μ*M perfusate was added with 5 minutes of reperfusion. In order to assess the infarct size, the isolated rat heart was reperfused for 2 hours after 30 minutes of ischemia. The slices were incubated in 1% *w*/*v* triphenyltetrazolium chloride (TTC, pH 7.4) for 15 min and then fixed in 10% formaldehyde. The Image-Pro Plus software (Media Cybernetics) was used to calculate the infarct area. The infarct area was expressed as a percentage of the LV area at risk.

### 2.4. *In Vivo* Myocardial I/R Model

Surgical ligation of the left coronary artery (LCA) was performed as described previously [17]. Mice were anesthetized with pentobarbital sodium (50 mg/kg) and ketamine (50 mg/kg) by intraperitoneal injection, followed by orally intubating and ventilating. The core body temperature is always maintained at 37°C. An internal sternotomy was then performed with electrocautery, and then, the proximal LCA was displayed and ligated. We investigated the magnitude of protection afforded by two different doses of pinocembrin (5 and 10 mg/kg i.v.). Accordingly, the mice received at random an intravenous injection of either saline, 5 mg/kg of pinocembrin, and 10 mg/kg of pinocembrin. These intravenous injections were performed during the last 5 min of ischemia, prior to reperfusion. After 30 minutes of coronary artery occlusion, the suture was cut and the blood vessels were allowed to reperfuse. After 24 hours of reperfusion, the mice were anesthetized with isoflurane. Transthoracic echocardiography was used to determine the left ventricular ejection fraction. After reperfusion, blood samples were taken followed by centrifugation at 3000 rpm for cardiac troponin T (cTnT) and lactate dehydrogenase (LDH) measurements. Measurement of an area at risk and infarct size was performed as reported previously.

### 2.5. Isolation and Culture of Primary Cardiomyocytes

Continuous enzymatic digestion and isolation were used to obtain neonatal rat and mouse cardiomyocytes [18, 19]. Neonatal rats were decapitated, and hearts were immediately placed in HBSS. The ventricle was taken and digested with trypsin and collagenase several times for 10 min at 37°C. Cardiomyocytes were suspended in sterile DMEM. The cells were preplated twice (37°C for 45 minutes) to reduce fibroblast contamination. Hypoxia/reoxygenation (H/R) was conducted as previously described in cardiomyocytes to simulate MI/R *in vivo* [20].

### 2.6. Measurement of LDH Release and cTnT Release

Necrotic cell death was assessed by the activity of the supernatant LDH, just like in previous studies [21]. The plasma cTnT level was used as an indicator of myocardial cell damage and was measured using a mouse cTnT ELISA kit (Wuhan Elabo Biotech Co., Ltd., China) according to the instructions.

### 2.7. Quantitative Real-Time PCR

Total RNA was extracted from the heart tissue using Trizol Reagent (Invitrogen, Carlsbad, CA, USA). Relative quantitation by real-time PCR was performed with SYBR Premix Ex Taq kits (TaKaRa) with the ABI PRISM 7900 System (Applied Biosystems). The primers targeting Gck, Galm, Gp1, Hk2, PFKFB3, Pdk1, Pdk2, Pdk3, Glut10, Glut4, Glut2, Glut1, Pdk4, Eno2, Bpgm, Aldoa, Aldob, Aldoc, and Pfk1 genes were list in Supplemental Table 1. GAPDH was used as internal normalization. The reactions were done at 95°C for 5 min followed by 40 cycles of 95°C for 30 s, 60°C for 30 s, and 72°C for 30 s.

### 2.8. Western Blot Analysis

Western blot was performed as described previously [22]. Left ventricles were homogenized, and the cells were directly lysed in ice-cold RIPA buffer. The samples were analyzed by SDS-PAGE. Transfer the protein to a polyvinylidene fluoride microporous membrane (Bio-Rad) with primary antibodies PFKFB3 (Abcam, USA; 1: 1000), HIF1*α* (CST, USA; 1: 1000), and anti-GAPDH (internal control; Kangcheng Co., Ltd., China; 1 : 8000). The bands were detected by horseradish peroxidase-coupled secondary antibodies (Cell Signaling Technology, 1 : 6000) and were visualized using the ECL detection kit (Amersham Pharmacia Biotech) and quantified with a video documentation system (Gel Doc 2000, Bio-Rad).

### 2.9. Generation and Administration of Adeno-Associated Virus (AAV)

Serotype 9 AAV vectors (AAV9) encoding shNC or shPFKFB3 (AAV9-shNC and AAV9–shPFKFB3) were prepared as previously described [23]. 3 × 10^11^ vg of AAV9-shNC or AAV9–shPFKFB3 was injected intravenously into tail veins as previously described [24] of 4-5-week-old male C57 mice. Sham or myocardial ischemia surgeries were conducted 4 weeks after AAV9 injection.

### 2.10. Seahorse Extracellular Flux Analyzer Assays

Cellular bioenergetics was measured using a Seahorse XFe24 extracellular flux analyzer in intact cardiomyocytes. We conducted glycolysis stress testing following the manufacturer's instructions as previously reported [25]. Glycolysis stress test: cells were incubated in glucose-free Seahorse assay media supplemented with 1 mM pyruvate at 37°C in an incubator without CO_2_ for 1 h prior to the assay. Injectors were loaded to add 20 mM glucose, 1 *μ*M oligomycin, and 100 mM 2 deoxy-glucose (2-DG), and glycolysis, glycolytic capacity, and glycolytic reserves were calculated as an extracellular acidification rate (ECAR).

### 2.11. Generation of Plasmids and Transfection

p-GL4-basic luciferase expression vectors containing various lengths of 5′-flanking regions from the human PFKFB3 gene promoter and an HIF1-luciferase reporter gene were prepared by gene synthesis by General Biol (Anhui, China). siRNAs for mouse HIF1*α* were obtained from RiboBio (Guangzhou, China). Transfection was performed with Lipofectamine 3000 (Thermo, MA, USA) following the manufacturer's protocol. Luciferase activities were measured by using a Dual-Luciferase® Reporter Assay System (Promega, USA).

### 2.12. Statistical Analysis

Statistical analysis was undertaken only for studies where each group size was at least *n* = 5 using software GraphPad Prism (v7, GraphPad Software, USA). Data were presented as means ± SEM. Comparisons between 2 groups were performed using unpaired, two-tailed *t* test, and ANOVA with *post hoc* Dunnett's test was used for among multiple groups. A *P* value < 0.05 was deemed statistically significant.

## 3. Results

### 3.1. Compound Pinocembrin Significantly Improved the Cardiac Function and Reduced Infarct Size after I/R *Ex Vivo*

**Figure 1:**
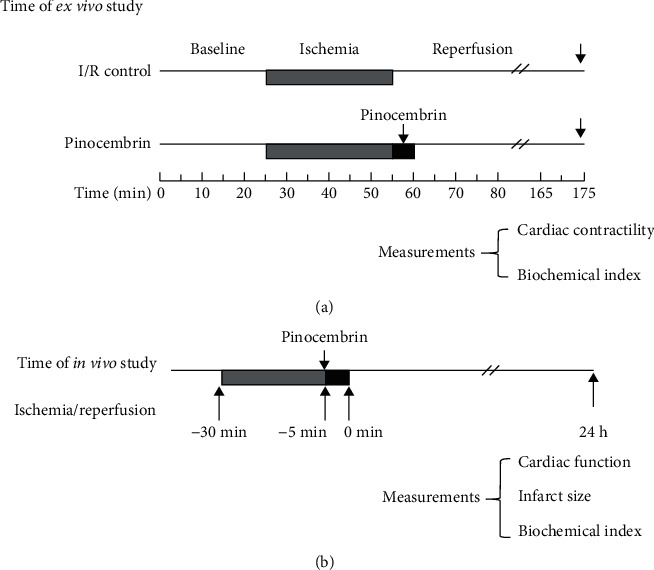
Experimental protocols to investigate the effects of pinocembrin on MI/R: (a) protocol of I/R ex vivo study (1); (b) protocol of MI/R in vivo study.

**Figure 2:**
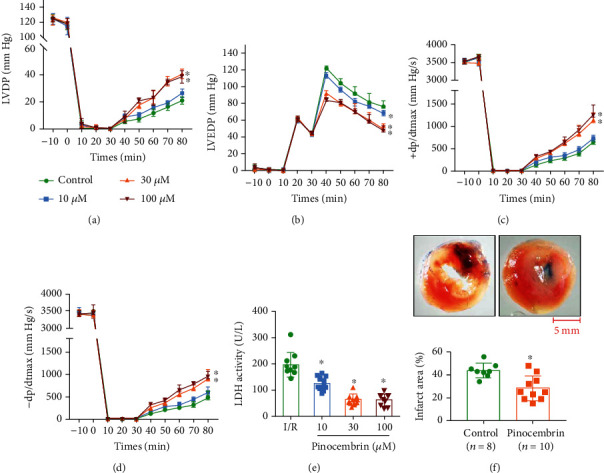
Compound pinocembrin significantly improves cardiac contractile recovery and reduces infarct size and LDH leakage after ischemia-reperfusion (I/R) *ex vivo*. (a–d) Time course of LVDP (a), LVEDP (b), and +dP/dtmax (c) and -dP/dtmax (d); *n* = 10 each. (e) Effect of pinocembrin on the LDH activity in the coronary effluent; *n* = 10 each. (f) Representative images and analysis of the infarct size in isolated I/R (30 min/2 h) hearts; *n* = 7 in the control group; *n* = 10 in the pinocembrin group. Data represent the mean ± SEM. ^∗^*P* < 0.05 versus I/R control group.

We perfused isolated rat hearts to explore the cardioprotective effects of pinocembrin against I/R injury. Ten to 100 *μ*M pinocembrin were delivered during the first 5 min of reperfusion (Figure 1(a)). During 45 min of reperfusion following 30 min ischemia, the contractile function of the left ventricle (LV) was significantly suppressed (Figures 2(a)–2(d)). Pinocembrin itself does not affect the heart rate during reperfusion (Supplementary Figure 1), while it dose-dependently improves the postischemic myocardial performance from 10 to 100 *μ*M (Figures 2(a)–2(d)).

Next, we explore whether pinocembrin improves cell survival during I/R via examining lactate dehydrogenase (LDH) release, an indicator of myocardial injury. Little LDH release was detected in the coronary efflux before ischemia, while LDH release was obviously induced at the end of reperfusion, while pinocembrin significantly inhibited the release of LDH from 10 to 100 *μ*M (Figure 2(e)). Consistently, pinocembrin significantly reduced the I/R-induced infarction after 2 h of reperfusion at the concentration of 30 *μ*M (Figure 2(f)). These results demonstrated that pinocembrin exhibits protective effects on cardiac I/R injury *ex vivo*.

### 3.2. Compound Pinocembrin Protects Hearts from Myocardial I/R Injury *In Vivo*

**Figure 3:**
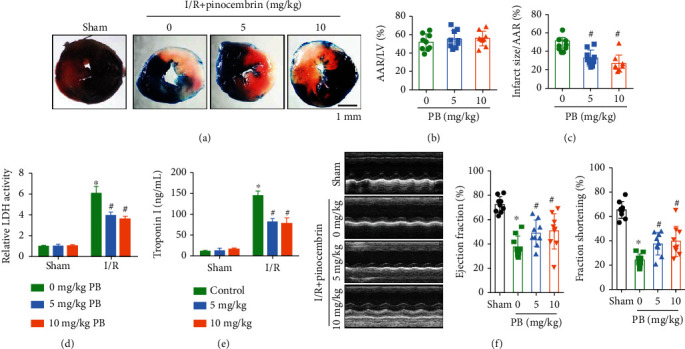
Pinocembrin protects from myocardial I/R (MI/R) injury in vivo in C57/BL6 mice. Wild-type (WT) mice were subjected to 30 minutes of the left anterior descending coronary artery (LAD) ligation, followed by 24 h of reperfusion, vehicle or pinocembrin intravenously (5 and 10 mg/kg) 5 min before reperfusion. (a) Representative TTC-stained LV transverse slices (scale bar, 1 mm). Areas stained dark blue, white, and red represented nonrisk, infarcted, and ischemic but noninfarcted zones, respectively. (b) Analysis of AAR (% total LV). (c) Analysis of myocardial infarct size (%AAR). (d, e) Plasma LDH activity and cTnI levels. (f) Representative images and analysis of left ventricular EF and FS, *n* = 9 each. Data represent the mean ± SEM. ^∗^*P* < 0.05 versus sham group. ^#^*P* < 0.05 versus I/R control group.

To explore the cardioprotective effects of pinocembrin on myocardial I/R injury *in vivo*, pinocembrin was intravenously injected into wild-type (WT) mice 5 minutes before the end of sustained ischemia (i.v., 5 mg/kg and 10 mg/kg), followed by reperfusion for 24 hours (Figure 1(b)). Evans-blue/TTC dye method was used to determine the infarct size. After reperfusion for 24 hours, no difference of area at risk (AAR) was observed between each group (Figures 3(a) and 3(b)). Nevertheless, compared with the I/R group, pinocembrin i.v. treatment significantly decreased the infarct size by 20% (Figures 3(a) and 3(c)). Besides, plasma levels of cTnI and LDH activity were markedly elevated during myocardial I/R, which were both suppressed with pinocembrin i.v. treatment (Figures 3(d) and 3(e)). Furthermore, the echocardiographic results showed that pinocembrin can significantly improve I/R-suppressed ejection fraction (EF) and fractional shortening (FS) (Figure 3(f)).

### 3.3. Protective Effects of Pinocembrin against H/R Induced Cardiomyocyte Injury

**Figure 4:**
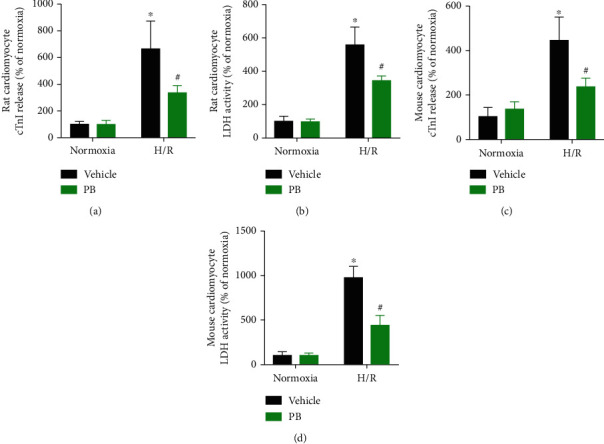
Protective actions of pinocembrin against cardiomyocyte injury responses in vitro. (a) Effect of pinocembrin (30 *μ*M, added during reperfusion) on rat cardiomyocyte cTnI release subsequent to H/R. (b) Effect of pinocembrin on rat cardiomyocyte LDH release subsequent to H/R. (c) Effect of pinocembrin on mouse cardiomyocyte cTnI release subsequent to H/R. (d) Effect of pinocembrin on mouse cardiomyocyte LDH release subsequent to H/R. *n* = 5 each. Data represent the mean ± SEM. ^∗^*P* < 0.05 versus normoxia with the vehicle group. ^#^*P* < 0.05 versus H/R with the vehicle group.

To determine whether pinocembrin confers cardioprotective effects through its direct action on the cardiomyocytes, isolated neonatal rat and mouse cardiomyocytes were subjected to H/R and applied 30 *μ*M pinocembrin during the onset of reperfusion. In accordance with the effects of pinocembrin on the myocardial I/R injury, simulated I/R-reduced cell viability was significantly improved by pinocembrin treatment (data not shown). Moreover, our data demonstrated that pinocembrin rescued cardiac troponin I (cTnI) release and LDH release postsimulated I/R *in vitro*, in both rat and mouse cardiomyocytes (Figures 4(a)–4(d)).

### 3.4. Pinocembrin Increases Glycolysis in Cardiomyocyte

**Figure 5:**
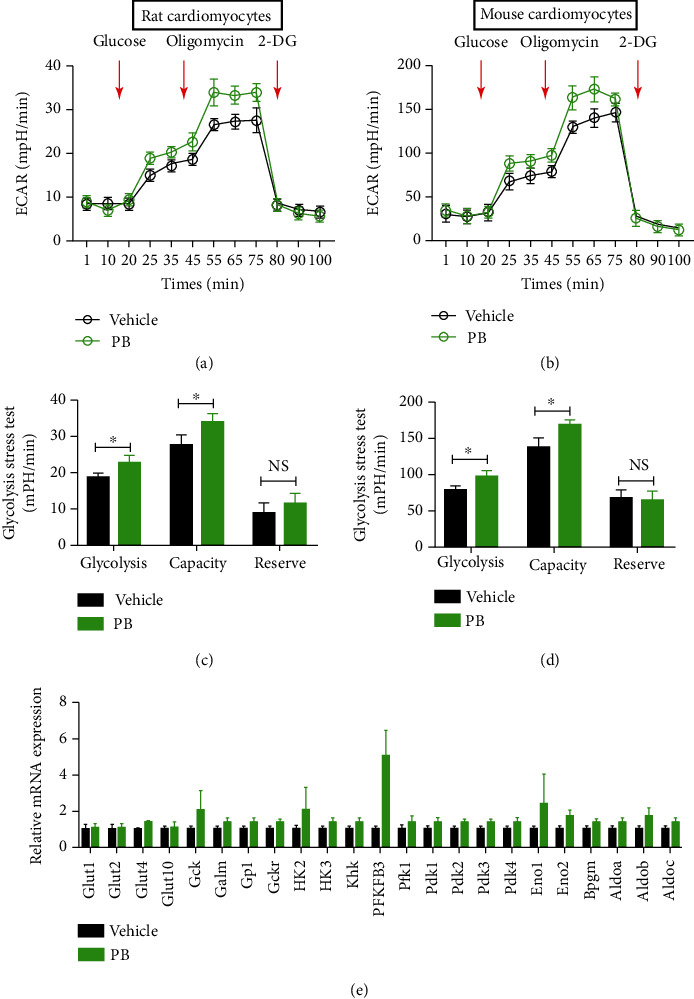
Pinocembrin increases glycolysis in primary cardiomyocytes. (a) Glycolysis stress test in rat cardiomyocytes measured as the ECAR by sequential injection of 20 mM glucose, 1 *μ*M oligomycin followed by 100 mM 2-deoxy-d-glucose (2-DG). (b) Quantitative analysis of glycolysis, glycolytic capacity, and glycolytic reserves in rat cardiomyocytes. (c) Glycolysis stress test in mouse cardiomyocytes measured as the ECAR by sequential injection of 20 mM glucose, 1 *μ*M oligomycin, and 100 mM 2-DG. (d) Quantitative analysis of glycolysis, glycolytic capacity, and glycolytic reserves in mouse cardiomyocytes pretreated with pinocembrin or control. (e) Mouse cardiomyocytes were treated with pinocembrin for 24 h, and then, the glycolysis-related genes were detected by qRT-PCR. *n* = 3/treatment in all Seahorse assays, each in triplicate. Data represent the mean ± SEM. ^∗^*P* < 0.05 indicates a significant difference. NS indicates no significant difference

During myocardial ischemia, enhanced glycolytic metabolism is essential for maintaining homeostasis of cardiomyocytes. In addition, previous studies reported that pinocembrin was involved in regulating glucose uptake in cancer cells [26]. Subsequently, we explored the effects of pinocembrin on cellular bioenergetics with the Seahorse extracellular flux analyzer and performed glycolysis stress tests to measure glycolysis and glycolytic capacity both in intact rat and mouse cardiomyocytes. Our results indicate that delivery with pinocembrin versus the control increased glycolysis by 21.4% (extracellular acidification rate (ECAR)) after H/R. Pinocembrin also increased glycolytic capacity in cardiomyocyte by 23.7% (Figures 5(a) and 5(b)).

To figure out the underlying mechanism of pinocembrin promoting myocardial glycolysis, mRNA expressions of glycolysis-related genes in cardiomyocytes after H/R were determined with qRT-PCR. As shown in Figure 5(c), pinocembrin significantly increased the expression of glycolysis-related genes, especially the PFKFB3 gene.

### 3.5. PFKFB3 Inhibition Alters Glycolysis and Abolished Pinocembrin-Induced Cardioprotection in Cardiomyocytes

**Figure 6:**
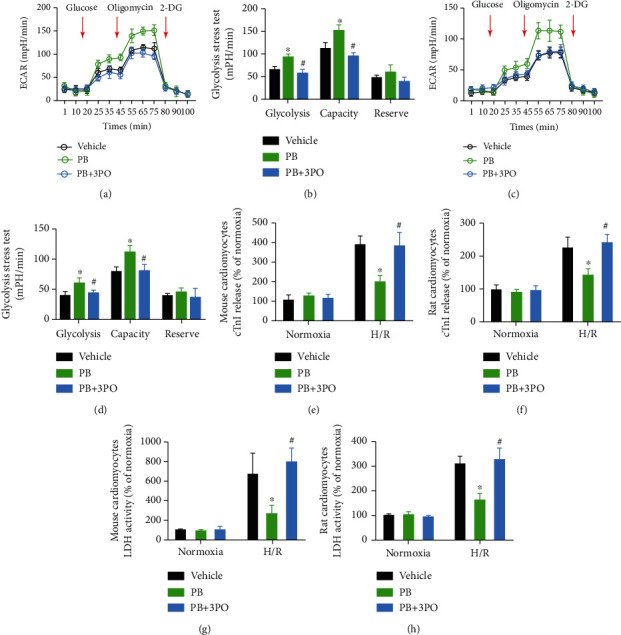
Pinocembrin protects cardiomyocytes from simulated I/R injury via enhancing glycolysis. (a–d) Treatment with PFKFB3 inhibitor, 3PO, results in a decline of glycolysis and glycolytic capacity in mouse and rat cardiomyocytes. *n* = 3/treatment in all Seahorse assays, each in triplicate. (e, f) Effect of PFKFB3 inhibitor, 3PO, on mouse and rat cardiomyocyte cTnI release subsequent to H/R. *n* = 5 each. (g, h) Effect of PFKFB3 inhibitor, 3PO, on mouse and rat cardiomyocyte LDH release subsequent to H/R. *n* = 5 each. Data represent the mean ± SEM. ^∗^*P* < 0.05 versus H/R with the vehicle group. ^#^*P* < 0.05 versus H/R with the PB group.

Next, we explored whether blockade of PFKFB3 inhibits cardiomyocyte glycolysis and abolishes pinocembrin-afforded cardioprotective effects. As shown in Figures 6(a)–6(d), exposure of cardiomyocyte to 10 *μ*M PFKFB3 inhibitor, 3PO remarkably reversed pinocembrin-enhanced glycolysis. What is more, inhibition of PFKFB3 resulted in a significant increase of cTnI release and LDH release post-H/R *in vitro* (Figures 6(e)–6(h)), suggesting that disruption of glucose metabolism leads to impaired cardioprotection of pinocembrin during H/R.

### 3.6. PFKFB3 Deficiency in Normal Mice Using AAV9 Abolished Pinocembrin-Afforded Cardioprotective Effects after MI/R

**Figure 7:**
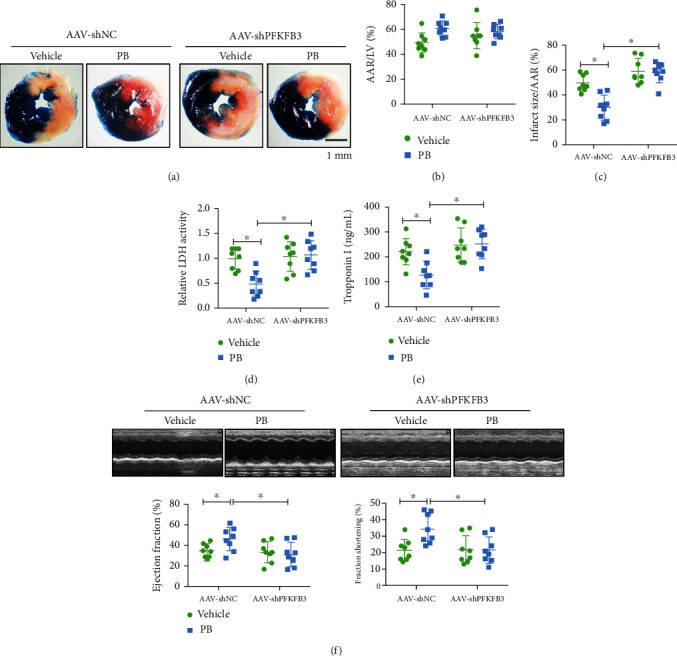
Knockdown of PFKFB3 in the myocardium using AAV9 impairs pinocembrin-induced cardioprotection after MI/R. (a) Representative infarct staining from AAV-shNC or AAV-shPFKFB3 mice treated with vehicle or pinocembrin. (b, c) AAR and infarct sizes from AAV-shNC or AAV-shPFKFB3 mice treated with vehicle or pinocembrin. (d, e) Plasma LDH activity and cTnI levels. (f) Representative echocardiographic images, assessment of LV EF and FS by echocardiography from AAV-shNC or AAV-shPFKFB3 mice treated with vehicle or pinocembrin. *n* = 8 each. Data represent the mean ± SEM. ^∗^*P* < 0.05, significantly different as indicated.

Since pinocembrin alleviated H/R-induced cardiomyocyte death by upregulating glycolysis via PFKFB3, we therefore evaluated the roles of PFKFB3 on myocardial I/R injury *in vivo*. To determine whether PFKFB3 regulation is directly involved in pinocembrin-related MI/R injury improvement, the WT mice were injected with AAV9 encoding PFKFB3 shRNA to knockdown endogenous PFKFB3. Intravenous injection of AAV9-shPFKFB3 on mice has successfully reduced the protein level of PFKFB3 in the heart (Supplementary Figure 2). Subsequently, mice were insulted with in situ myocardial ischemia-reperfusion to assess the functional role of myocardial-specific PFKFB3 in pinocembrin-afforded cardioprotection. Myocardial injury was measured by infarct size area, serum cTnT levels, and LDH activity. Exposure of mice infected with AAV9-shPFKFB3 to MI/R presented larger infarct sizes and enhanced release of troponin I and LDH (Figures 7(a)–7(c)). More importantly, the deletion of myocardial PFKFB3 abolished pinocembrin-conferred protective effects on those indexes. Furthermore, the pinocembrin-improved EF and FS were also abolished by PFKFB3 deficiency with AAV9-shPFKFB3 (Figure 7(d)). Collectively, these data confirm that pinocembrin confers cardioprotective effects by enhancing cardiomyocyte glycolysis through activation of PFKFB3.

### 3.7. HIF1*α* Is a Key Transcription Factor Driving Pinocembrin-Induced PFKFB3 Expression

**Figure 8:**
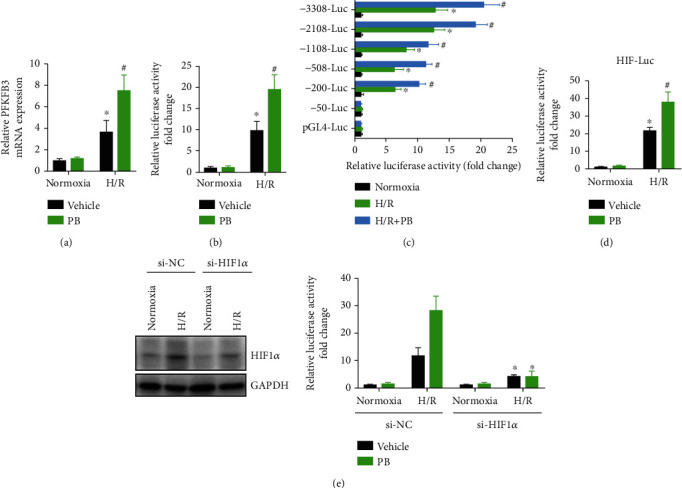
Pinocembrin promotes PFKFB3 expression through activating transcription factor HIF1*α*. (a) Cardiomyocytes were treated with pinocembrin and subsequent to H/R, and then, PFKFB3 expression was detected by qRT-PCR. ^∗^*P* < 0.05 versus normoxia with the vehicle group. ^#^*P* < 0.05 versus H/R with the vehicle group. (b) Cardiomyocytes were transiently cotransfected with a pGL4-basic luciferase expression vector, containing 3,308 bp of the mouse PFKFB3 gene promoter. Cells were exposed to H/R with or without pinocembrin. Transfections were performed in triplicate. ^∗^*P* < 0.05 versus normoxia with the vehicle group. ^#^*P* < 0.05 versus H/R with the vehicle group. (c) Cardiomyocytes were transiently transfected with pGL4-basic luciferase expression vectors containing various lengths of the PFKFB3 gene promoter 5′-flanking region, and cells were treated with pinocembrin subsequent to H/R. ^∗^*P* < 0.05 versus normoxia with the vehicle group. ^#^*P* < 0.05 versus H/R with the vehicle group. (d) Transcriptional activity of reporter constructs carrying binding sites for HIF1*α* transfected in cardiomyocytes insulted with H/R. ^∗^*P* < 0.05 versus normoxia with the vehicle group. ^#^*P* < 0.05 versus H/R with the vehicle group. (e) Analysis of PFKFB3 promoter activity in cardiomyocytes transfected with control, HIF1*α*-specific siRNA. *n* = 5 each. Data represent the mean ± SEM. ^∗^*P* < 0.05 versus corresponding si-NC group.

Pinocembrin notably promoted PFKFB3 gene expression in cardiomyocytes exposed to H/R (Figure 8(a)). To explore the underlying mechanisms responsible for the increased expression of PFKFB3 in cardiomyocytes induced by pinocembrin, we examined the activity of the mouse PFKFB3 promoter both in the absence or in the presence of pinocembrin. We transfected cardiomyocytes with a construct in which luciferase expression was driven by a 3,308 bp fragment from the mouse PFKFB3 promoter. As shown in Figure 8(b), pinocembrin by itself exerted little effect on PFKFB3 promoter activity, but it cooperated with H/R to increase luciferase activity (Figure 8(b)). Furthermore, we studied the DNA regions required for pinocembrin to increase PFKFB3 promoter activity by using a series of promoter constructs containing successive deletions from the 5′ end. As shown in Figure 8(c), 5′ deletion mutants revealed that sequences between -50 and -200 and -1108 and -2108 from the transcription start site are essential for the induction of the PFKFB3 gene promoter by pinocembrin.

HIF1*α* is considered to be an important regulator of glycolysis, and its target genes include PFKFB3 and glucose transporter-1 (GLUT1) [27, 28]. Also, pinocembrin increased the expression of HIF1*α*, which are key transcription factors for PFKFB3 expression (data not shown). To identify transcription factor HIF1*α* that could modulate PFKFB3 induction by pinocembrin, reporter constructs carrying binding sites for HIF1 were transfected into cardiomyocytes, which were stimulated afterward with pinocembrin. The results showed that the activity of HIF1 reporter constructs increased upon activation with H/R, and this activity was enhanced by the pinocembrin (Figure 8(d)). Moreover, we observed that reduced expression of HIF1*α* with specific siRNA greatly decreased PFKFB3 induction by pinocembrin during H/R, corroborating its key role in pinocembrin-induced PFKFB3 expression (Figures 8(e) and 8(f)). Together, our data demonstrates that HIF1*α* is a key transcription factor driving pinocembrin-induced PFKFB3 expression during H/R.

## 4. Discussion

In the present study, we observed that glycolysis functionally mediated the cardioprotective responses of pinocembrin. We demonstrated that (i) pinocembrin delivered at the onset of reperfusion (postconditioning) significantly improved postischemic myocardial function and reduced infarct size after I/R *ex vivo*; (ii) pinocembrin significantly protected mouse hearts from acute myocardial I/R injury *in vivo*; (iii) these protections are partially related to enhanced glycolysis by pinocembrin in the I/R cardiomyocytes; and (iv) the cardioprotective effects of pinocembrin are mediated by the activation of PFKFB3. These results extend previous findings indicating the cardioprotection of pinocembrin against I/R injury and reveal the new mechanisms of pinocembrin in the cardioprotection.

It would be an attractive treatment principle to reduce the infarct size through pharmaceutical intervention to assist classic reperfusion intervention [29]. Pinocembrin is a potential cardiovascular drug with potential neuroprotective effects on transient and long-term ischemic stroke in rats [30]. Also, a previous study indicated that the administration of pinocembrin before myocardial ischemia improved LV function [7]. Pharmacological postconditioning is easier to implement and has therapeutically potential in both clinical and experimental settings, which avoids the potential injury induced by ischemic conditioning, and therefore has good clinical application prospects [31]. Therefore, our study aims to explore the cardioprotective effects of pinocembrin postconditioning. The e*x vivo* results showed that pinocembrin from 10 to 100 *μ*M delivered at the first 5 minutes of reperfusion remarkably improved postischemic myocardial function and attenuates cell death in a concentration-dependent manner. Furthermore, the *in vivo* mouse myocardial I/R injury model was prepared and pinocembrin postconditioning was fulfilled by an intraperitoneal injection of pinocembrin (5 mg/kg and 10 mg/kg body weight) 5 min before reperfusion. Cardiac function, serum LDH activity and cTnT content, and infarct size were significantly improved with pinocembrin treatment. The cardioprotective effects of pinocembrin are consistent with other reports. This suggests that pinocembrin could be a candidate compound that may be used in the clinical treatment of myocardial infarction.

Various metabolic abnormalities occur during myocardial I/R, such as increased fatty acid oxidation and decreased glucose oxidation [32]. This phenomenon is related to the uncoupling of mitochondrial respiration, increased proton leakage, ROS formation, and, more importantly, increased myocardial oxygen consumption [33]. During myocardial I/R, the metabolic shift aimed at increasing glucose oxidation has proved to be beneficial. Although some therapeutic strategies have tried to reverse this metabolic imbalance, there is still no approved treatment regimen so far [34–36]. In addition, strategies aimed at increasing clinical glucose consumption have different results and have not yet reached routine clinical practice. Looking for drugs that can safely induce the transfer of cellular energy metabolism and a better understanding of the protective mechanisms of increased glucose oxidation may facilitate transfer to the clinic. Our results show that increasing glycolysis is responsible for pinocembrin-induced protective effects. This is consistent with previous studies that metabolic shift towards increased glycolysis protects the heart from I/R injury [13]. Moreover, PFKFB3 expression is notably upregulated during I/R, which directs cellular glucose metabolism from PPP to aerobic glycolysis [37]. Our data have shown that PFKFB3 was significantly upregulated with pinocembrin treatment and specific inhibitor of PFKFB3 abolished pinocembrin-afforded protective effects in cardiomyocytes. Most importantly, knockdown of PFKFB3 in the myocardium using AAV9 reversed the cardioprotection of pinocembrin postconditioning. Previous studies have shown that the main reason responsible for the protection offered by the metabolic shift is the fact that glycolysis is able to produce two molecules of ATP without the need for oxygen, so that uncoupling between mitochondrial full glucose oxidation and glycolysis leads to increased cardiac efficiency [38]. However, potential protective mechanisms of the metabolic shift are largely unknown. Further studies are needed to investigate how pinocembrin regulate the glycolysis and search for its putative downstream targets.

## 5. Conclusions

In summary, our findings demonstrate that compound pinocembrin exhibits significant protective effects on cardiac I/R injury when delivered at the beginning of or before reperfusion in *ex vivo* rat and *in vivo* mouse models through enhancing glycolysis. Pinocembrin may be considered as an effective lead compound for large animal experiments and is expected to be used in clinical research of acute myocardial infarction.

## Data Availability

The data used to support the findings of this study are available from the corresponding author upon request.

## Supplementary Materials

Supplementary Materials Supplemental Figure 1: effects of pinocembrin on the heart rate and coronary flow (CF) in isolated rat hearts subjected to 30 min no-flow global ischemia followed by 45 min of reperfusion. Supplemental Figure 2: (A) schematic time scale and experimental strategy to evaluate the effect of PFKFB3 expression on pinocembrin-afforded cardioprotection. Adeno-associated virus of serotype 9 (AAV9) encoding nontarget (NC) or shRNA for PFKFB3 was injected intravenously into tail veins of mice (3^∗^1011 vg) at week 0. Four weeks later, sham or myocardial ischemia surgeries were conducted. (B) Protein levels of PFKFB3 in myocardium treated with control shRNA (AAV-shNC) or FetB shRNA (AAV-shPFKFB3). Data represent the mean ± SEM. ^∗^*P* < 0.05 versus indicated group. Supplemental Table 1: primers used in this study.
