# Supplementary material for: Cardioprotective Natural Compound Pinocembrin Attenuates Acute Ischemic Myocardial Injury via Enhancing Glycolysis
Source: Oxid Med Cell Longev. 2020 Oct 15;2020:4850328. doi: 10.1155/2020/4850328 (PMC7644300; doi:10.1155/2020/4850328)
Supplement: Supplementary Materials — Supplemental Figure 1: effects of pinocembrin on the heart rate and coronary flow (CF) in isolated rat hearts subjected to 30 min no-flow global ischemia followed by 45 min of reperfusion. Supplemental Figure 2: (A) schematic time scale and experimental strategy to evaluate the effect of PFKFB3 expression on pinocembrin-afforded cardioprotection. Adeno-associated virus of serotype 9 (AAV9) encoding nontarget (NC) or shRNA for PFKFB3 was injected intravenously into tail veins of mice (3∗1011 vg) at week 0. Four weeks later, sham or myocardial ischemia surgeries were conducted. (B) Protein levels of PFKFB3 in myocardium treated with control shRNA (AAV-shNC) or FetB shRNA (AAV-shPFKFB3). Data represent the mean ± SEM. ∗P < 0.05 versus indicated group. Supplemental Table 1: primers used in this study. [file 4850328.f1.docx]

**Cardioprotective natural compound pinocembrin attenuates acute ischemic myocardial injury via enhancing glycolysis**

Short Running Title: Pinocembrin, glycolysis and cardioprotection

Yanjun Zheng^1,2^, Guoqing Wan^1,2^, Bo Yang^1^ ,Xuefeng Gu^1,2^ and Jingrong Lin^3^

^1^Shanghai University of Medicine & Health Sciences Affiliated Zhoupu Hospital, Pudong New Area, Shanghai, 201318, China.

^2^Shanghai Key Laboratory of Molecular Imaging, Shanghai University of Medicine and Health Sciences, Shanghai 201318, China.

^3^Department of Hypertension, Ruijin Hospital, Shanghai Institute of Hypertension, Shanghai Jiao Tong University School of Medicine, Shanghai, China.

Leading Correspondence: Jingrong Lin, PhD

Department of Hypertension, Ruijin Hospital, Shanghai Institute of Hypertension, Shanghai Jiao Tong University School of Medicine, 197 Ruijin 2^nd^ Rd, Shanghai 200025, China.

Tel: +86 21 64370045

E-mail: [jingrong.lin@163.com](mailto:jingrong.lin@163.com)

Correspondence: Xuefeng Gu, PhD

Shanghai University of Medicine & Health Sciences Affiliated Zhoupu Hospital, Pudong New Area, Shanghai, 201318, China.

Tel: +86 21 65883218

E-mail: [xfgu@sumhs.edu.cn](mailto:xfgu@sumhs.edu.cn)


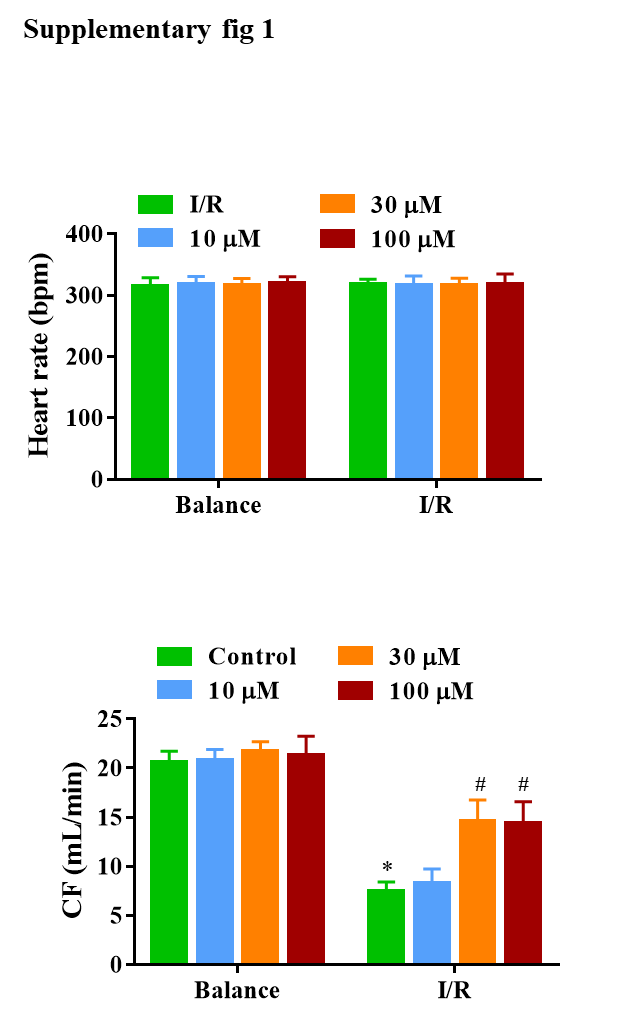


**Supplemental Figure 1.** Effects of pinocembrin on the heart rate and coronary flow (CF) in isolated rat hearts subjected to 30 min no-flow global ischemia followed by 45 min of reperfusion. (A-B) Analysis of Heart rate (A) and CF (B) at preischemia (Balance) and 45 min of reperfusion (I/R). n = 4 each. *P < 0.05, **P < 0.01, ***P < 0.001 vs. preischemic controls or indicated ones. n=10. Data represent the mean ± SEM. **P*<0.05 versus Balance with control group. ^#^*P*<0.05 versus I/R with control group.


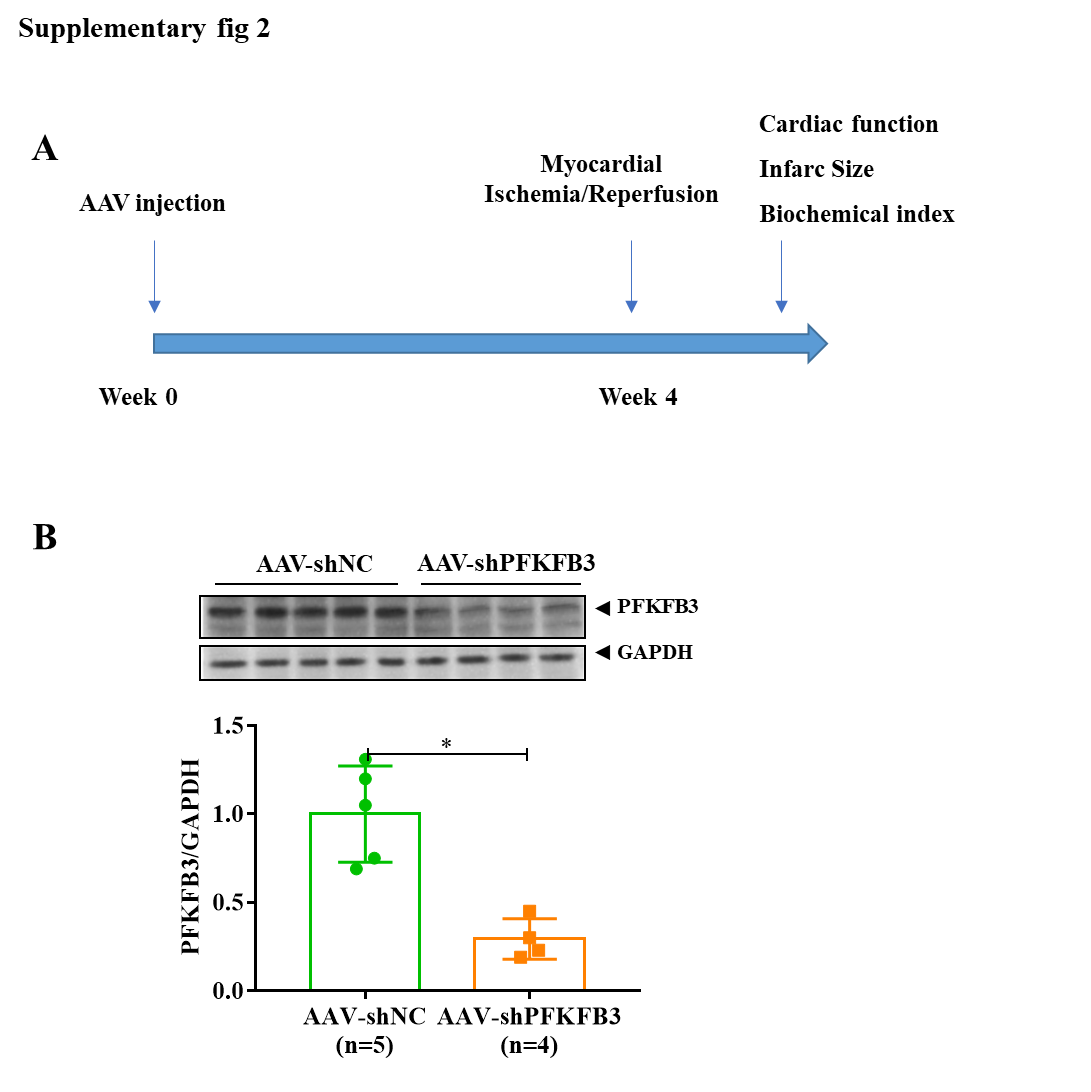


**Supplemental Figure 2. A,** Schematic time scale and experimental strategy to evaluate the effect of PFKFB3 expression on pinocembrin-afforded cardioprotection. Adeno-associated virus of serotype 9 (AAV9) encoding non-target (NC) or shRNA for PFKFB3 were injected intravenously into tail veins of mice (3*10^11^ vg) at week 0. Four weeks later, Sham or myocardial ischemia surgeries were conducted. B. Protein levels of PFKFB3 in myocardium treated with control shRNA (AAV-shNC) or FetB shRNA (AAV-shPFKFB3). Data represent the mean ± SEM. **P*<0.05 versus indicated group.

**Supplemental Table 1.** Primers used in this study

| ***Targets*** | ***Primer sequence (5’-3’)*** |
| --- | --- |
| GAPDH | F- GTGGCAAAGTGGAGATTGTTG |
|  | R- CTCCTGGAAGATGGTGATGG |
| Gck (glucokinase) | F- AGGAGGCCAGTGTAAAGATGT |
|  | R- CTCCCAGGTCTAAGGAGAGAAA |
| Galm (galactose mutarotase) | F- CTGCACGATCACTGCTCTG |
|  | R- GGTAACCTTCCAATTCCGCAA |
| Gp1(GDP-D-glucose phosphorylase 1) | F- CCAGGAGGATCTCGTTGGC |
|  | R- AGAGTCGAAACGGGATAGTGG |
| Hk2 (hexokinase 2) | F- ATGATCGCCTGCTTATTCACG |
|  | R- CGCCTAGAAATCTCCAGAAGGG |
| Pfkfb3 | F- CAACTCCCCAACCGTGATTGT |
|  | R- TGAGGTAGCGAGTCAGCTTCT |
| Pdk1(pyruvate dehydrogenase kinase isoenzyme 1) | F- GGACTTCGGGTCAGTGAATGC |
|  | R- TCCTGAGAAGATTGTCGGGGA |
| Pdk2 | F- AGGGGCACCCAAGTACATC |
|  | R- TGCCGGAGGAAAGTGAATGAC |
| Pdk3 | F- TCCTGGACTTCGGAAGGGATA |
|  | R- ACCTCTCTCATGGTGTTAGCC |
| Glut10 (glucose transporter 10) | F- GGGCCTGACCTTCGGATATG |
|  | R- GCTCCTGTTCGAGGCAACT |
| Glut4(glucose transporter 4) | F-ACACTGGTCCTAGCTGTATTCT |
|  | R-CCAGCCACGTTGCATTGTA |
| Glut2(glucose transporter 2) | F-TCAGAAGACAAGATCACCGGA |
|  | R-GCTGGTGTGACTGTAAGTGGG |
| Glut1(glucose transporter 1) | F-GCAGTTCGGCTATAACACTGG  R-GCGGTGGTTCCATGTTTGATTG |

| ***Targets*** | ***Primer sequence (5’-3’)*** |
| --- | --- |
| Pdk4 | F- AGGGAGGTCGAGCTGTTCTC |
|  | R- GGAGTGTTCACTAAGCGGTCA |
| Eno2(enolase 2) | F- AGGTGGATCTCTATACTGCCAAA |
|  | R- GTCCCCATCCCTTAGTTCCAG |
| Bpgm(2,3-bisphosphoglycerate mutase) | F- GGACCAGAAACTTAACAACGACG |
|  | R- CATAGTGACGCTCATTCAGACG |
| Aldoa (aldolase A) | F- AGTCCACCGGAAGCATTGC |
|  | R- CAGCCCCTGGGTAGTTGTC |
| Aldob (aldolase B) | F- GAAACCGCCTGCAAAGGATAA |
|  | R- GAGGGTCTCGTGGAAAAGGAT |
| Aldoc (aldolase C) | F- AGAAGGAGTTGTCGGATATTGCT |
|  | R- TTCTCCACCCCAATTTGGCT |
| Pfk1(phosphofructokinase 1) | F- CATCGCCGTGTTGACCTCT |
|  | R- CCCGTGAAGATACCAACTCGG |
